# Plumage iridescence is associated with distinct feather microbiota in a tropical passerine

**DOI:** 10.1038/s41598-019-49220-y

**Published:** 2019-09-09

**Authors:** Veronika Gvoždíková Javůrková, Erik D. Enbody, Jakub Kreisinger, Kryštof Chmel, Jakub Mrázek, Jordan Karubian

**Affiliations:** 1 Faculty of Agrobiology, Food and Natural Resources, Department of Animal Science, Czech University of Life Sciences, Kamýcká 129, 165 00 Prague, Suchdol Czech Republic; 2 Institute of Vertebrate Biology, Czech Academy of Sciences, Květná 8, 603 65 Brno, Czech Republic; 3 Department of Ecology and Evolutionary Biology, Tulane University, New Orleans, LA USA; 4 Faculty of Science, Department of Zoology, Charles University, Viničná 7, 128 44 Prague, Czech Republic; 5 Faculty of Science, Department of Zoology, University of South Bohemia, Branišovská 1760, 370 05 České Budějovice, Czech Republic; 6 Institute of Animal Physiology and Genetics, Czech Academy of Sciences, Vídeňská 1083, 160 00 Prague-Krč, Czech Republic

**Keywords:** Microbial ecology, Coevolution, Environmental microbiology

## Abstract

Birds present a stunning diversity of plumage colors that have long fascinated evolutionary ecologists. Although plumage coloration is often linked to sexual selection, it may impact a number of physiological processes, including microbial resistance. At present, the degree to which differences between pigment-based vs. structural plumage coloration may affect the feather microbiota remains unanswered. Using quantitative PCR and DGGE profiling, we investigated feather microbial load, diversity and community structure among two allopatric subspecies of White-shouldered Fairywren, *Malurus alboscapulatus* that vary in expression of melanin-based vs. structural plumage coloration. We found that microbial load tended to be lower and feather microbial diversity was significantly higher in the plumage of black iridescent males, compared to black matte females and brown individuals. Moreover, black iridescent males had distinct feather microbial communities compared to black matte females and brown individuals. We suggest that distinctive nanostructure properties of iridescent male feathers or different investment in preening influence feather microbiota community composition and load. This study is the first to point to structural plumage coloration as a factor that may significantly regulate feather microbiota. Future work might explore fitness consequences and the role of microorganisms in the evolution of avian sexual dichromatism, with particular reference to iridescence.

## Introduction

Avian plumage is a unique integumentary structure that is critical for multiple functions including flight^1–3^, thermoregulation^4–6^, and socio-sexual communication^7,8^. Feather coloration is a product of deposited pigments (e.g. carotenoids, melanins and psittacofulvins) responsible for pigment coloration^9,10^, feather integumentary nanostructures responsible for structural coloration^11–13^, or a combination of both^14–17^. Elaboration of feather coloration generated by combinations of these factors is considered to be primarily driven by sexual selection^18^. In this context, studies have demonstrated that variation in pigment-based and structural plumage coloration is under sexual selection by advertising quality and/or reproductive success^16,19–22^. Carotenoid-based coloration is more prone to diet, foraging strategy and an individual’s physiological state^23–25^. On the contrary, association between melanin-based plumage coloration and reproductive parameters and/or survival in birds is species-specific and dependent on adaptation to local environmental conditions^26,27^ that may include interactions with omnipresent microorganisms.

Feathers are subject to exposure to the external environment and host diverse microbial communities^28–31^ including antibiotic compounds-producing microorganisms^31^, pathogens^32^ or feather-degrading bacteria^29,33^. The latter can deteriorate feather structure^34,35^ and negatively affect signaling function of both pigment based and structural plumage coloration^36–38^. In addition, plumage bacterial load may significantly impair individual immunity and fitness^39–41^. However, experimental evidence suggests that feather pigments, particularly melanins and also parrot psittacofulvins, significantly improve resistance of feathers against bacterial degradation^34,42,43^. Moreover, feather melanization was found to inhibit attachment and colonization of keratinolytic bacterium *B*. *licheniformis* on black- and white-striped feathers^44^. Prevention of feather bacterial degradation via increased deposition of melanins into the feathers is one functional explanation in several studies that have documented more melanized individuals in colder, wetter and more densely vegetated habitats^45–48^. Yet, a study comparing feather microbial load and diversity in individuals adopting different melanin-based plumage phenotypes in natural population of birds is lacking.

Recent studies have highlighted the role of structural based plumage coloration such as iridescence, in the evolution of avian plumage coloration and dichromatism^13,49–51^. Iridescent feathers (which seem to be ancestral in birds^52^) have decreased hydrophobicity^53^ and are more sensitive to bacterial degradation^54^, which provide a potential mechanism for honest signaling of individual quality. However, increased plumage bacterial load has been shown to diminish brightness of iridescent neck feathers in pigeons^38^. Moreover, iridescent plumage phenotypes show greater diversity in tropical and sub-tropical species^12,49,55–58^ that are exposed to higher and more diversified microbial loads^59,60^. It follows, therefore, that apart from a protective role of feather pigments, birds may have developed other defense mechanisms for preventing detrimental effects of external parasites including microorganisms on feather wear^61^.

To date, no study has investigated the relationship between iridescent plumage phenotype and indices of feather microbiota in free living populations of birds. In the present study, we used molecular approaches to investigate feather microbial load and diversity in two populations of a tropical passerine bird, the White-shouldered Fairywren (*Malurus alboscapulatus*), of New Guinea that vary in the extent of melanin-based and structural plumage coloration both between populations and between sexes. No other study has investigated the consequences of feather microbial load and diversity for divergent plumage phenotypes in melanin-based and structural coloration for a natural bird population. We leverage this unique variation to ask how feather microbial load and diversity varies between plumage phenotypes and discuss mechanistic underpinnings and consequences for plumage signaling and evolution.

## Material and Methods

**Figure 1:**
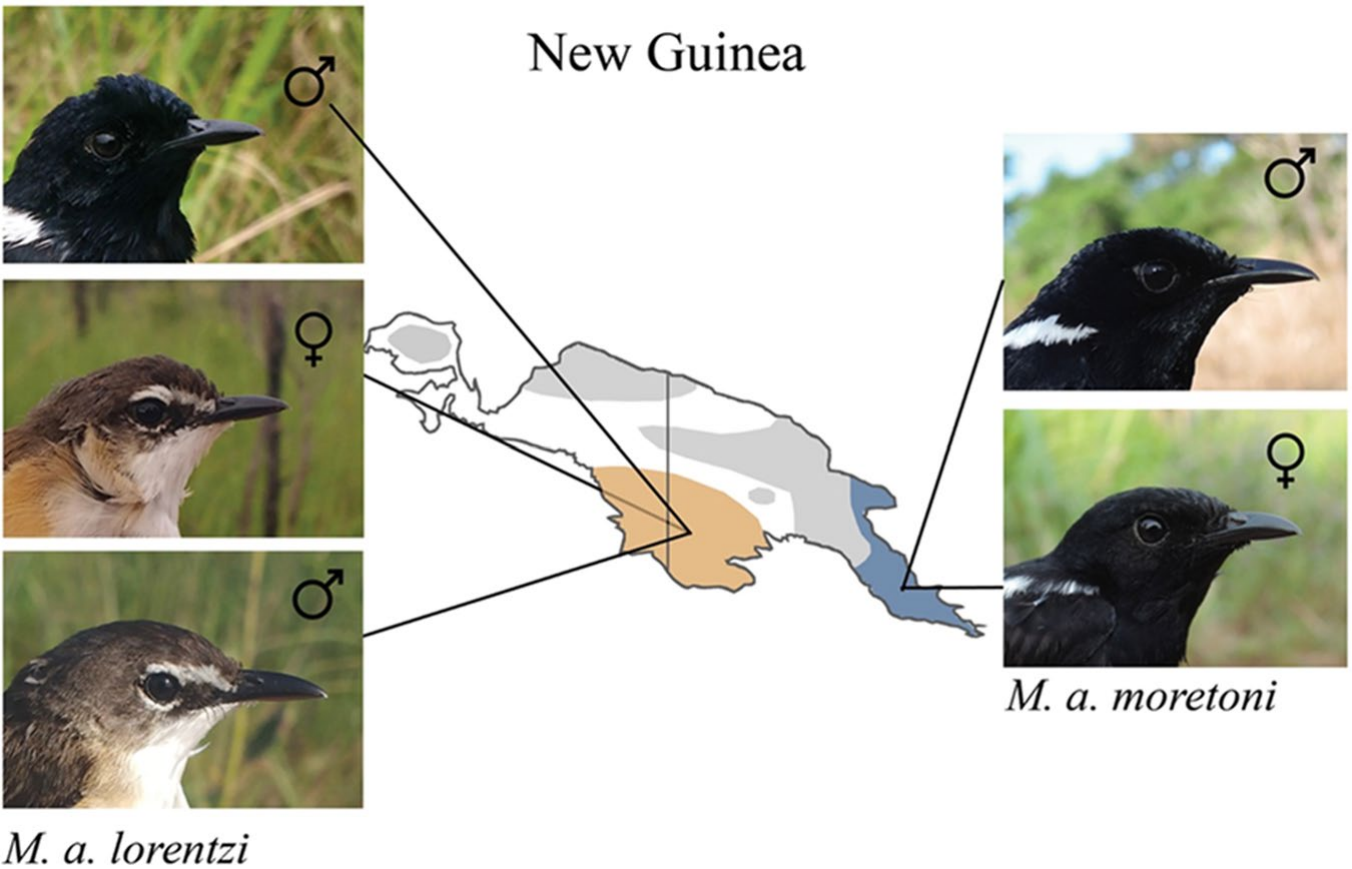
Photographs and localities (i.e. populations) of White-shouldered Fairywren subspecies included in this study and sampled in Papua New Guinea. Within the *M*. *a*. *lorentzi* subspecies, we sampled white chest feathers from brown females and first-year males and black chest feathers from iridescent males, while within *M*. *a*. *moretoni* subspecies, black chest feathers from ornamented black males with iridescent plumage and black females with matte plumage were sampled (photographs: Erik D. Enbody).

We analyzed feather microbial load, diversity and community profile in two subspecies of White-shouldered Fairywren (family Maluridae, Meyer, 1874), a socially breeding, tropical, insectivorous passerine endemic to grassland environments^62^ in New Guinea. Both subspecies are sexually dichromatic, yet their plumage phenotypes differ in melanin-based and structural coloration. While females and first year males of subspecies *M*. *a*. *lorentzi* are brown dorsally and white ventrally^63^ (“brown individuals” hereinafter), adult males are black with white shoulder patches including an iridescent blue satin sheen. In contrast, males and females of *M*. *a*. *moretoni* exhibit cryptic sexual dichromatism as they are similar in appearance and both are black with white shoulder patches, yet black females are matte, lack the male’s iridescent blue satin sheen and have lower barbule density compared to iridescent black males^64^ (Fig. 1).

All individuals were sampled under permit 2194 issued by the Australian Bird and Bat Banding Scheme and protocols were reviewed under the Tulane University IACUC number 0395 and all experiments were performed in accordance with relevant institutional guidelines and regulations.

### Study area and sampling procedure

We collected chest contour feathers from 24 individuals of White-shouldered Fairywren that were mist-netted during February-March 2016 in two provinces of Papua New Guinea. Specifically, white chest feathers from two first-year males and three females of the subspecies *M*. *a*. *lorentzi* were sampled in Western Province, Papua New Guinea (141°19′E, 7°35′S, 10–20 m ASL; Fig. 1). Black chest feathers from four matte black females of the *M*. *a*. *moretoni* subspecies were sampled in Milne Bay Province, Papua New Guinea (150°30′E, 10°15′S, 0–20 m ASL; Fig. 1) and fifteen iridescent black males were sampled between two allopatric subspecies populations; Western Province (*n* = 11), and Milne Bay Province (*n* = 4).

Chest contour feathers (approx. 10–15) were plucked from each individual using sterile forceps and immediately placed into a sterile tube filled with 1 mL of 96% sterile-filtered ethanol. To avoid contamination, feathers for microbiological analyses were collected directly from individuals trapped in mist net (i.e. prior to handling). After removing the individuals from the mist net, age and sex of each individual was assigned. We also collected a blood sample from each brown individual of *M*. *a*. *lorentzi* and stored it in Longmire’s lysis buffer for subsequent genetic determination of sex.

### Sex identification

We assigned sex in the field to individuals with cloacal protuberances, brood patches, or sex-specific plumage phenotypes. As first year males and adult females of *M*. *a*. *lorentzi* are apparently identical in appearance (Fig. 1), we used molecular markers to sex the five brown individuals sampled in this population. We extracted DNA from blood samples using a DNeasy blood and tissue kit (Qiagen) and amplified a sex-specific intron within the CHD gene using primers 2550F/2718R^65^. We ran CHD intron fragments through electrophoresis using a 2% agarose minigel and stained with SYBR Safe DNA gel stain (Life Technologies). Bands were scored visually following^66^, using positive controls to confirm accuracy.

### Analyses of feather microbial load and diversity

#### DNA extraction

To measure feather microbial load and diversity, microbial genomic DNA was isolated from feather samples stored in ethanol. See Supplementary Material and Methods for complete DNA extraction protocol.

#### Quantification of feather microbial load

To analyze feather microbial load, we used quantitative PCR targeting 16S rRNA in extracted microbial DNA from feather samples using a LightCycler®480 Instrument (Roche, Mannheim, Germany). See Supplementary Material and Methods for complete qPCR amplification conditions.

#### Analysis of feather microbial diversity and community profiling

Denaturing Gradient Gel Electrophoresis (DGGE) was used to assess feather microbial diversity and community profile. See supplementary Material and Methods for detailed DGGE protocol.

DGGE gel image was processed in BioNumerics software v 7.6 (Applied Maths, Belgium) for normalization, bands detection and band matching table construction. Bands were detected using the band-search algorithm with densitometric curves describing the optical density along each lane and enabling background subtraction. Band detection criterions was set as 5% relative to maximum densitometric value of lane to eliminate uncertain bands. Then, for each sample running on DGGE gel, the number of Operational Taxonomic Units (OTUs) and Shannon-Wiener diversity index (H_sw_) were calculated based on equation:

$$(Hsw)=\mathop{\sum }\limits_{i=1}^{n}-\frac{hi}{H}\,log\frac{hi}{H}$$

where *n* is the total number of bands, *h*_i_ the intensity of the individual band *i* and *H* the total intensity of all bands in a profile, were calculated. Band matching tables were computed using band densitometric peak height, peak surface, and 2D band areas. We measured the relative OTUs abundances with band matching optimization and tolerance set as 0.5%. This semi-quantitative band matching table was used for the computation of Bray-Curtis and Jaccard distance matrices using the R package phyloseq.^67^.

#### Taxonomy of feather microbial communities

To identify the most representative and abundant bacteria within White-shouldered Fairywren plumage microbial communities, the 12 most pronounced DGGE bands (see Supplementary Fig. S1) were cut out of the stained polyacrylamide gels by the sterile scalpel in a dark room on a UV light-box. DNA was eluted by the addition of 100 µL of sterile dH_2_O and centrifuged at 10,000 rpm for 10 minutes. Then, 2 µL of this solution with primers FP341 (5′-CCTACGGGAGGCAGCAG-3′) and RP534 (see above) was used for amplification under the PCR-DGGE program^68^. The resulting PCR products were cleaned with QIAquick PCR purification kit (Qiagen, Germany) and sequenced using standard Sanger methods from both sides (SeqMe service, Czech Republic).

Taxonomy assignment of obtained representative sequences were done with the RDP classifier^69^ by combining the Greengenes database (version 13_8)^70^ with 80% posterior probability limit and Geneious Prime (version 2019.0.4). To assess prevalence of the most representative OTUs among plumage phenotypes, presence vs. absence data for all 12 sequenced DGGE bands within White-shouldered Fairywren indiviudals was extracted from the normalised DGGE gel (see Supplementary Fig. S1) using Bionumerics software v 7.6 (Applied Maths, Belgium). Heatmap showing the phylogeny and prevalence of the 12 representative OTUs (i.e. DGGE bands) within White-shouldered Fairywren plumage phenotypes were generated using the R packages ggtree^71^ ape^72^ and ggplot2^73^.

### Statistics

All statistical analyses were performed in RStudio (Version 1.1.453)^74^. We used Analysis of Variance (ANOVA) models with log-transformed 16S rRNA copy number per mg of feather and Shannon diversity index as response variables to evaluate factors affecting differences in feather microbial load, and feather microbial alpha diversity in White-shouldered Fairywren subspecies, respectively. Plumage phenotype (brown vs. black), sex (male vs. female) nested within plumage phenotype, and age coded as binary variable (0 = first-year birds, 1 = birds older than first-year) were included as categorical explanatory variables in each ANOVA model. Tukey HSD post-hoc tests were used for multiple comparison of significant effects and their means between tested categories.

Iridescent black males were sampled in two geographically distinct populations; Milne Bay and Western (see Material and Methods for details), and we used Welch’s Two Sample t-test due to unequal variance in the case of microbial load, and Student’s t-test in the case of alpha diversity testing between-population effect on feather microbial load and diversity.

To assess factors responsible for divergence in similarity (i.e. β-diversity) of feather microbial communities among White-shouldered Fairywren individuals, we used a PERMANOVA permutation test (R package vegan, function: adonis) by fitting a linear model based on both Bray-Curtis and presence/absence version of Jaccard similarity coefficients including plumage phenotype (brown vs. black) sex (male vs. female) nested within plumage phenotype and age as response variables. Due to unbalanced sampling design, we also assessed heterogeneity of variance (i.e. inter-individual variation of feather microbiota composition) between plumage phenotypes using the betadisper function^75^. Principal Coordinates Analysis (PCoA) was used to visualize among-sample divergence in composition of feather microbial communities.

## Results

### Feather microbial load

**Figure 2:**
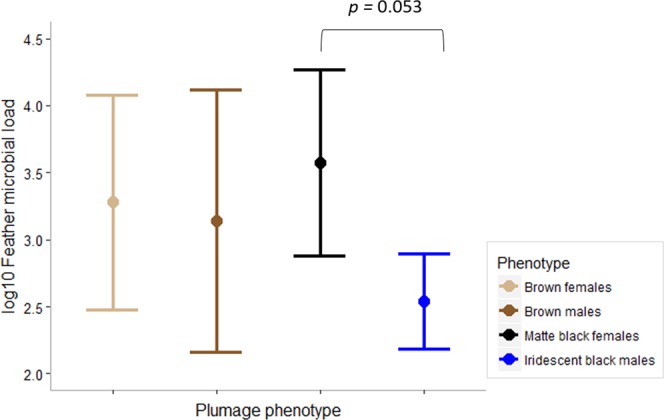
Differences (mean ± 95% CI) in feather microbial load (log_10_ 16S rRNA copy numbers per mg of feather) between White-shouldered Fairywren plumage phenotypes. Significant differences are based on Tukey’s HSD.

We found sex nested within plumage phenotype as significant predictor of White-shouldered Fairywren microbial load (ANOVA: *F*_(2,21)_ = 3.856, *p* = 0.038), with iridescent black males tended to have lower feather microbial loads than did matte black females (Tukey’s HSD test: *p* = 0.053; Table S1, Fig. 2). However, neither iridescent black males nor matte black females significantly differed in feather microbial load compared to brown individuals (Table S1, Fig. 2). Moreover, there was no effect of individual’s age on feather microbial load (ANOVA: *F*_(1,22)_ = 0.252, *p* = 0.621).

We found no differences in microbial loads of iridescent black males sampled in the two geographically distinct populations (Welch Two sample t-test: *t* = −1.398, *df* = 8.825, *p* = 0.196).

### Feather microbial diversity and taxonomic profile

**Figure 3:**
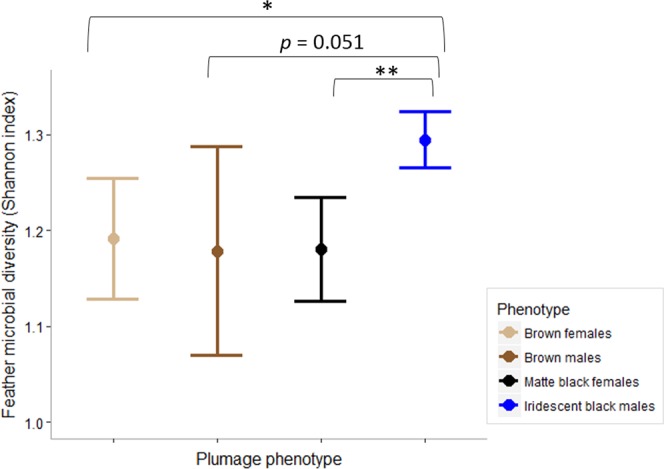
Differences (mean ± 95% CI) in feather microbial α-diversity (Shannon-Wiener index) between White-shouldered Fairywren plumage phenotypes. Significant differences are based on Tukey’s HSD (statistical significance: *p < 0.05, **p < 0.01).

Alpha diversity of feather microbial communities varied within sex nested in plumage phenotypes (ANOVA: *F*_(2,21)_ = 11.555, *p* < 0.001) with iridescent black males having significantly more diversified microbial communities than matte black females (Tukey’s HSD test: *p* = 0.001, Table S2, Fig. 3), brown females (Tukey’s HSD test: *p* = 0.017; Table S2, Fig. 3) and brown males where the effect was marginally non-significant (Tukey’s HSD test: *p* = 0.051; Table S2, Fig. 3). Effect of age on feather microbial alpha diversity was not significant (ANOVA: *F*_(1,22)_ = 1.668, *p* = 0.213). In addition, we did not find between-population differences in feather microbial alpha diversities between iridescent black males sampled in two geographically distinct populations (Student’s Two sample t-test: *t* = −1.375, *df* = 12, *p* = 0.194).

**Figure 4:**
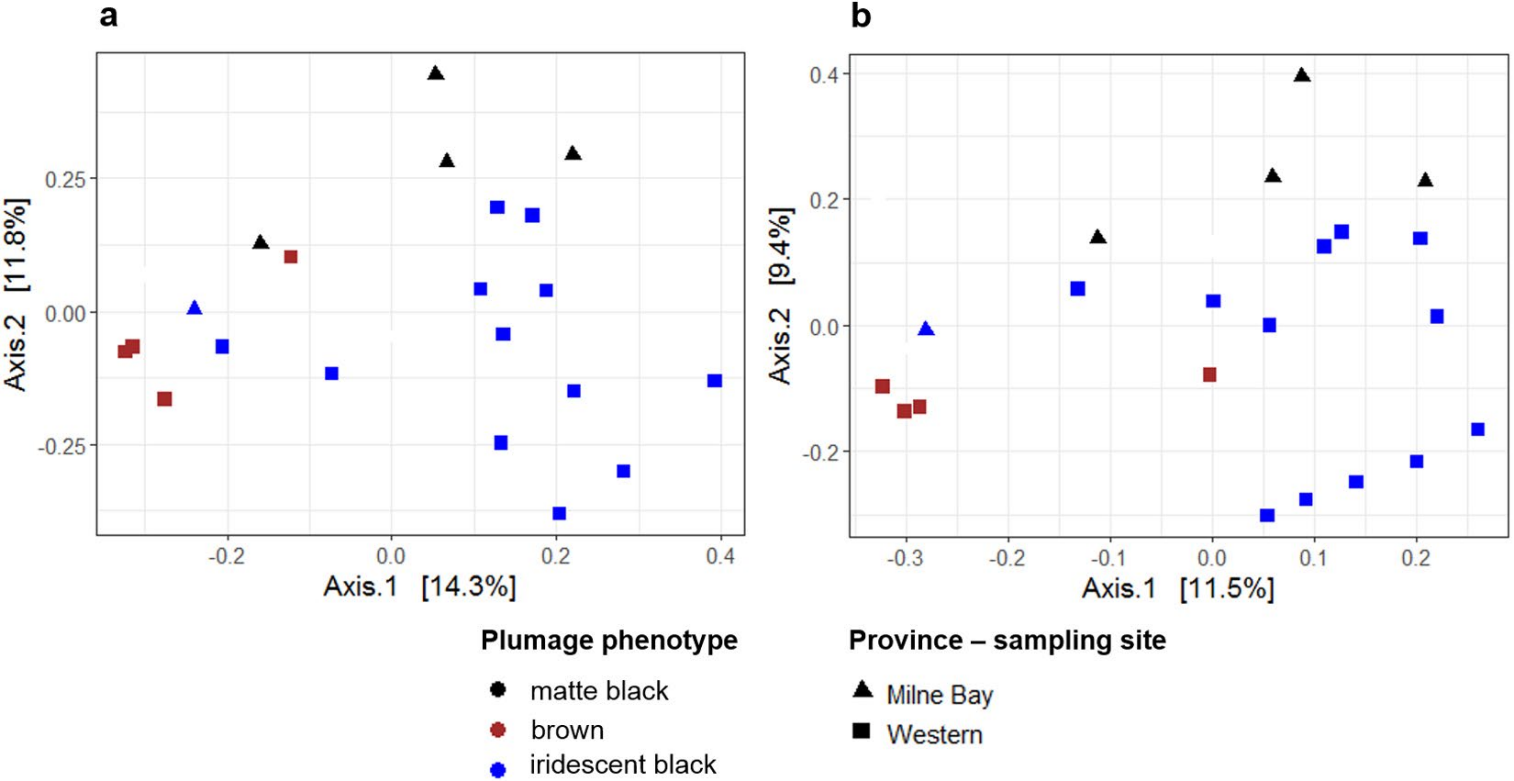
Principal Coordinates Analysis (PCoA) for among-sample divergence in composition (β-diversity) of White-shouldered Fairywren feather microbial communities based on (**a**) Bray-Curtis and (**b**) Jaccard dissimilarities. Different colors denoted plumage phenotypes and different shapes geographically distinct provinces (i.e. sampling localities).

Similarity (i.e. *β*-diversity) of feather microbial communities between White-shouldered Fairywren individuals was best explained by sex nested in plumage phenotype (PERMANOVAs: Bray-Curtis - 23% variance explained, *F* = 1.571, *p* = 0.001; Jaccard - 21% variance explained, *F* = 1.380, *p* = 0.002) with age having no significant effect (PERMANOVAs: Bray-Curtis - 10% variance explained, *F* = 1.361, *p* = 0.054; Jaccard - 9% variance explained, *F* = 1.147, *p* = 0.135). Heterogeneity of inter-individual variance among plumage phenotypes was significant for Bray-Curtis (betadisper: *F* = 26.123, *p* < 0.001) as well as Jaccard (betadisper: *F* = 37.400, *p* < 0.001). PCoA ordination shown that composition of feather microbial communities of iridescent black males varied from microbial communities of matte black females and brown individuals (Fig. 4a,b). PCoA visualization of between-population microbial community divergence showed no apparent dissimilarities in feather microbial community profiles of iridescent black males sampled in geographically distinct populations (Fig. 4a,b).

**Figure 5:**
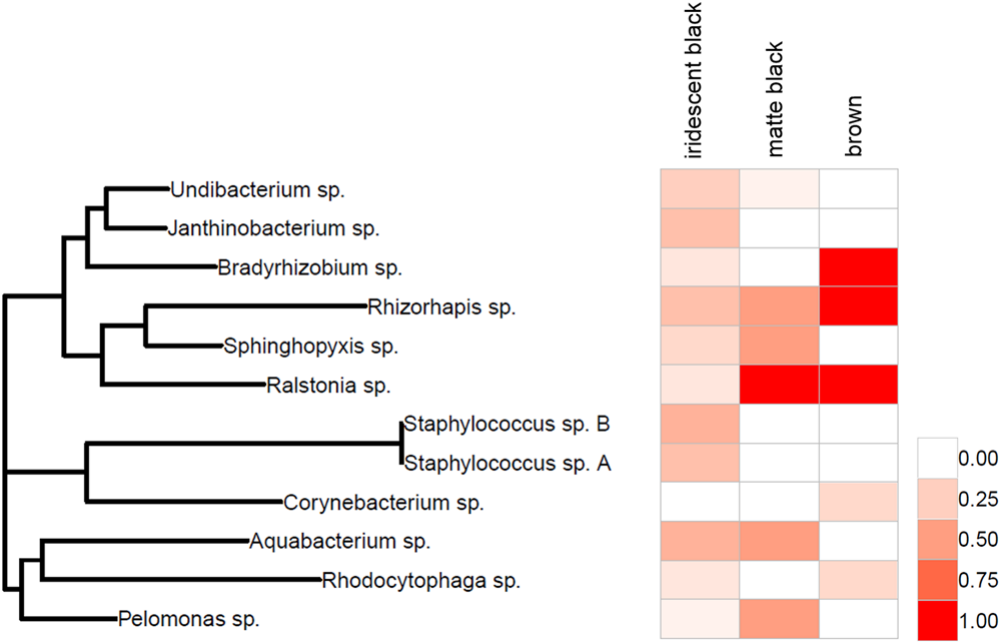
Heatmap showing taxonomic assignment and prevalence (%) of the most representative microbial genera detected in feather of White-shouldered Fairywren with different plumage phenotypes.

Taxonomic composition of the most representative feather microorganisms was dominated by the phyla *Betaproteobacteria*, *Alphaproteobacteria*, *Firmicutes*, *Actinobacteria* and *Bacteroidetes*. Prevalence of the most representative bacterial genera differed among plumage phenotypes, with brown individuals dominated by the genera *Bradyrhizobium*, *Rhizorhapis* and *Ralstonia* while black individuals hosted other genera with various prevalence depending on presence/absence of structural coloration (Fig. 5). A detailed taxonomy of the most representative bacterial genera found in White-shouldered Fairywren plumage and their prevalence among plumage phenotypes is presented in Fig. 5.

## Discussion

We show that the presence or absence of iridescent plumage, not melanized plumage per se, was associated with differences in feather microbiota in free-living populations of a tropical bird. Iridescent black males had the lowest feather microbial load, the highest microbial diversity and harbored a distinct microbial community relative to brown and matte black individuals of either sex. Our findings regarding feather microbial load are inconsistent with a recent study investigating bacterial load on ornamental throat feathers in free-living population of spotless starling (*Sturnus unicolor*), which documented higher bacterial load on iridescent feathers compared to un-ornamented adjacent or female feathers^54^.

Most existing research has shown that feather microbial diversity and community structure are primarily driven by horizontal transmission of microbes from the environment^31,76^. However, species-specific feather microorganisms that are able to produce antimicrobial substances^31^, or particular chemical substances contained in preen gland secretions^77–79^ may also affect feather microbiota diversity. Our data show that iridescent individuals originating from two geographically (>1000 km apart) and ecologically distinct populations^63^ do not differ in feather microbial diversity and harbor similar microbial communities on their feathers. Due to the similarity in microbial communities between individuals living in different environments, it is unlikely that horizontal transmission of microbes from the environment drive differences we observe in microbiota communities. Instead, chemical composition of preen gland secretions or physical properties of iridescent feathers based on UV reflectance and absorbance of solar radiation may be more important contributors to feather microbiota diversity and community structure in iridescent individuals.

We found no evidence that feather melanization impacts White-shouldered Fairywren feather microbiota. These findings did not necessarily exclude the hypothesis that melanins play a protective role against bacterial degradation of plumage^34,35,46,48^, as we did not directly test changes in degradability of differently melanized White-shouldered Fairywren feathers. Existing evidence suggests a preferential colonization and attachment of bacteria on white (i.e. non-melanized) compared to black (i.e. fully melanized) feathers or feather parts^44,80^, which we did not observe in our study system. A negative correlation between feather melanization and preening effort has been shown in barn owls^81^, suggesting that it is possible that brown individuals balance the feather microbiota via increased preening effort compared to matte black individuals that may comparatively invest less into the preening.

White-shouldered Fairywren individuals with iridescent feathers tended to have reduced feather microbial load compared to non-ornamented individuals. One possible explanation for this is differences in investment for plumage maintenance. Presently, we have no observational data proving that iridescent White-shouldered Fairywren individuals invest more into preening, but there is evidence that ornamented males of the Red-backed Fairywren (*Malurus melanocephalus*), the White-shouldered Fairywren’s sister species, preen at higher rates than do unornamented males (J. Karubian, unpublished data). In other avian species, studies have documented that preening behavior can significantly reduce feather microbial load^38,82^, enhance feather condition including waterproofness^83^, or increase feather visual signaling properties^84,85^. There is also evidence for associations between degree of feather microbial load and preen gland size^86–88^ supporting significant role of preen gland and its secretions in alterations of feather microbiota. Furthermore, allopreening (e.g. preening between mates) is important for maintaining social bonds across the genus *Malurus*^62^ and may be a mechanism through which male iridescent plumages are maintained by reducing microbial load. In this sense, our findings may be consistent with the “attractive preening” hypothesis, which suggests that ornamental iridescent plumage is linked with increased investment in plumage maintenance via preening^89^, which may come with a high energetic costs^39,90^ and may thus reflect bearer quality^91^.

However, it is certainly possible that other factors may drive this pattern. For example, iridescent plumage in this system differs in terms of nanostructure from non-iridescent plumage^64^, which may in turn influence many physical properties, including solar reflectivity^92^. It has been hypothesized that iridescent nanostructuring of feathers might reduce heat loss of colorful sexually selected pigment-based coloration by reflecting solar energy^92^ and iridescent feathers often have reflectance peaks in UVA and UVB spectrum^93,94^, which may inactivate or be lethal for most microorganisms^95–97^. Consequently, the solar heat absorption properties of iridescent feathers might have temperature-dependent effects on microorganisms present on feathers. Some of the bacterial genera detected in our study (*Staphylococcus*, *Aquabacterium*) and having different prevalences among plumage phenotypes, have been found in digestive tract of feather mites^98^. Consequently, another explanation is that feather mites may act as effector symbionts able to digest and thus selectively affect (based on plumage phenotype) feather microbiota^99^, which has been found in other species^98^. Feather mites have been detected on White-shouldered Fairywrens (E. Enbody, unpublished data), but further testing is needed to evaluate the interplay between feather mites and microbiota in this system.

Our observation of distinct microbiota communities and abundance between feather types suggests an overlooked role for structural coloration in complex plumage evolution and for the wild microbiome in host evolution. A better understanding of the proximate mechanisms behind the documented association between iridescent plumage phenotype and feather microbiota diversity, particularly in relation to preening gland secretions chemistry and preening behavior, is a priority for future research into the feather microbiome and plumage evolution.

## Supplementary information

Supplementary Information

## Data Availability

The nucleotide sequence data reported are uploaded in the GenBank database under the submission Numbers: MK215669, MK215670, MK215671, MK215672, MK215673, MK215674, MK215675, MK215676, MK215677, MK215678 and MK215679. The datasets generated during and/or analyzed during the current study are available from the corresponding author on reasonable request.
