# Supplementary material for: Plumage iridescence is associated with distinct feather microbiota in a tropical passerine
Source: Sci Rep. 2019 Sep 9;9:12921. doi: 10.1038/s41598-019-49220-y (PMC6733896; doi:10.1038/s41598-019-49220-y)
Supplement: Supplementary file 1 — Supplementary Information [file 41598_2019_49220_MOESM1_ESM.pdf]

ELECTRONIC SUPPLEMENTARY INFORMATION

Plumage iridescence is associated with distinct feather microbiota in tropical passerine

Veronika Gvoždíková Javůrková [1, 2]*, Erik D. Enbody [3], Jakub Kreisinger [4], Kryštof Chmel [4, 5], Jakub Mrázek [6], Jordan Karubian [3]

Supplementary information files:

1. Supplementary Material and Methods
2. Supplementary Figure S1
3. Supplementary Table S1
4. Supplementary Table S2

1. Supplementary Material and Methods

Analyses of feather microbial load and diversity

DNA extraction

Tubes containing contour feather samples in 96% ethanol were centrifuged at 20.000 x g for 20 minutes (Eppendorf 5424, Eppendorf, Germany) to pellet free microbial cells and feathers. Then, 800 µl of ethanol supernatant was removed from each tube and the remaining ethanol was evaporated at 56°C using thermo-shaker (BioSan TS-100, BioSan, Latvia). From prepared feathers and microbial cells in tubes, genomic DNA was extracted using the RTP® Bacteria DNA Mini kit (STRATEC Molecular GmbH, Berlin, Germany) following the isolation kit´s Protocol 5 (Isolation of microbial DNA from tissue biopsies). All samples manipulations and

isolation steps were performed by one person (VGJ) and under sterile condition in a laminar flow hood at non-invasive lab of the Institute of Vertebrate Biology, the Czech Academy of Sciences.

The weights of particular feather samples used for DNA extraction were obtained after DNA isolation. Immediately after DNA extraction, each feather sample was washed three times with 1mL of distilled water to remove residues of lysis buffer and dried at 95 °C on thermo-shaker. Then, feather dry mass of each sample was measured with a precision of 0.01mg using lab scale (KERN, ABT 120-5DM). Initial feather mass of each sample was then used for calculation of the total 16S rRNA copy number per mg of feather.

Quantification of feather microbial load

The KAPA SYBR® FAST for LightCycler®480 mastermix (Sigma Aldrich) and the universal Eubacteria primer set, including forward primer 27F (5′-AGAGTTTGATCCTGGCTCAG -3′) and reverse primer 534R (5′- ATTACCGCGGCTGCTGG -3′), were used for qPCR amplification[68]. Quantitative PCR amplification efficiency was checked for various primer concentrations and annealing temperatures. Optimal amplification conditions were achieved with 130 nM final concentration for each primer and an annealing temperature of 50 °C for a given primer set. The PCR reaction was performed in triplicate on a 96-well Hard-Shell®480 PCR (CLR/WHT) plate (Bio-Rad, USA) using a total volume of 15 µL, containing 7.5 µL of KAPA SYBR® FAST for LightCycler®480 mastermix, 6.1 µL of Microbial DNA-Free Water (Qiagen, USA), 0.195 µL of each primer at concentrations of 10 µM and 1 µL of 10-fold diluted DNA template. Plate was sealed with Microseal® C Film (Bio-Rad, USA). Reaction conditions for DNA amplification were: one pre-incubation cycle at 95 °C for 3 min followed by 40 amplification cycles at 95 °C for 10 sec., 50 °C for 25 sec., and 72 °C for 1 sec. To determine specificity of amplification, gel electrophoresis of amplified products together with analysis of

products´ melting curves were performed. A melting curve was obtained by slow heating at 2.2 °C/s increments from 65 °C to 97 °C, with fluorescence collection at 0.5 °C intervals. Serial dilutions (10^{-3} to 10^{-9}) of synthetized gBlock® 1555 bp fragment of *Bacillus subtilis* 16S rRNA (Integrated DNA Technologies, USA) were used as standard. A calibration curve was constructed and used for quantification of feather microbial load which was related to initial feather mass and expressed as number of 16S rRNA copies per mg of feather. Due to ubiquity of *B. subtilis* and genus *Bacillus* in bird´s plumage[28,48,100] and known *B. subtilis* 16S rRNA copy number variation which is intermediate compared to other bacteria[101], we decreased potential under/over estimation of 16S rRNA copy number in our samples using the gBlock® 16S rRNA fragment of *B. subtilis* as a standard. Efficiency, slope and amplification factor for qPCR run were: 86%; - 3.72 and 1.86, respectively.

Analysis of feather microbial diversity and community profiling

Twenty one DNA samples of 13 iridescent black males, 4 matte black females, and 4 brown individuals (3F:1M) were analyzed by targeting 16S rRNA gene amplification with universal microbial primers 338GC (5’-CGCCCGCCGCGCCCCGCGCCCGGCCCGCCGCCGCCGCACTCCTACGGGAGGCAGCAG - 3’) and RP534 (5’- ATTACCGCGGCTGCTGG - 3’). PCR assay was performed with the following amplification program: 3 minutes of denaturation step at 94°C, 35 cycles consisting of 1 min at 94°C, 30 seconds at 55°C, 1 minute at 72°C, and the final elongation step at 72°C for 10 minutes[68]. The PCR mixture contained 2 µL of DNA template, 0.5 µL of each primer (10 µM), 15 µL of OneTaq PCR Master Mix (Biotech, Czech Republic) and 12 µL of sterile H_2O. All 30 µL of PCR mixture was used in the DGGE analysis. Products from PCR were then processed by DGGE on the DCodeTM Universal Mutation Detection System (Bio-Rad, USA) on 9% polyacrylamide gel with 35 - 60% denaturing gradient. The

electrophoresis was realized in 7 L of 1x TAE buffer for 18 hours at 55 V and 60°C[102]. Thereafter, gels were stained in 50 mL of 1x TAE with SYBRr Green I dye (0.001%) for 30 minutes and visualized by UV light on Vilber Lourmat System (France).

References

28. Shawkey, M. D., Mills, K. L., Dale, C. & Hill, G. E. Microbial diversity of wild bird feathers revealed through culture-based and culture-independent techniques. *Microb. Ecol.* **50**, 40–47, https://doi.org/10.1007/s00248-004-0089-4 (2005).

48. Burtt, E. H. & Ichida, J. M. Gloger's rule, feather-degradlng bacteria, and color variation among song sparrows. *Condor* **106**, 681-686, https://doi.org/10.1650/7383 (2004).

68. Muyzer, G., Dewaal, E. C. & Uitterlinden, A. G. Profiling of complex microbial-populations by Denaturing Gradient Gel-Electrophoresis analysis of polymerase chain reaction-amplified genes-coding for 16S ribosomal-RNA. *Appl.Environ. Microbiol.* **59**, 695–700 (1993).

100. Burtt, E. H. & Ichida, J. M. Occurrence of feather-degrading bacilli in the plumage of birds. *Auk* **116**, 364–372, https://doi.org/10.2307/4089371 (1999).

101. Vetrovsky, T. & Baldrian, P. The Variability of the 16S rRNA Gene in Bacterial Genomes and Its Consequences for Bacterial Community Analyses. *PLoS One* **8**, https://doi.org/10.1371/journal.pone.0057923 (2013).

102. Fischer, S. G. & Lerman, L. S. Length-independent separation of dna restriction fragments in 2-dimensional gel-electrophoresis. *Cell* **16**, 191–200, https://doi.org/10.1016/0092-8674 (79)90200-9 (1979).

2. Supplementary Figure S1

Figure S1. Denaturing Gradient Gel Electrophoresis (DGGE) profiles of White-shouldered Fairywren plumage microbiota of four matte black females (**lines 1-4**), four brown individuals (**lines 5-8**) and thirteen iridescent black males (**lines 9-21**). Twelve most pronounced bands (i.e. OTUs) that were sequenced and used for taxonomic assignment of the most representative White-shouldered Fairywren plumage microorganisms and their prevalence among plumage phenotypes presented in Fig. 5 are denoted by red arrows.

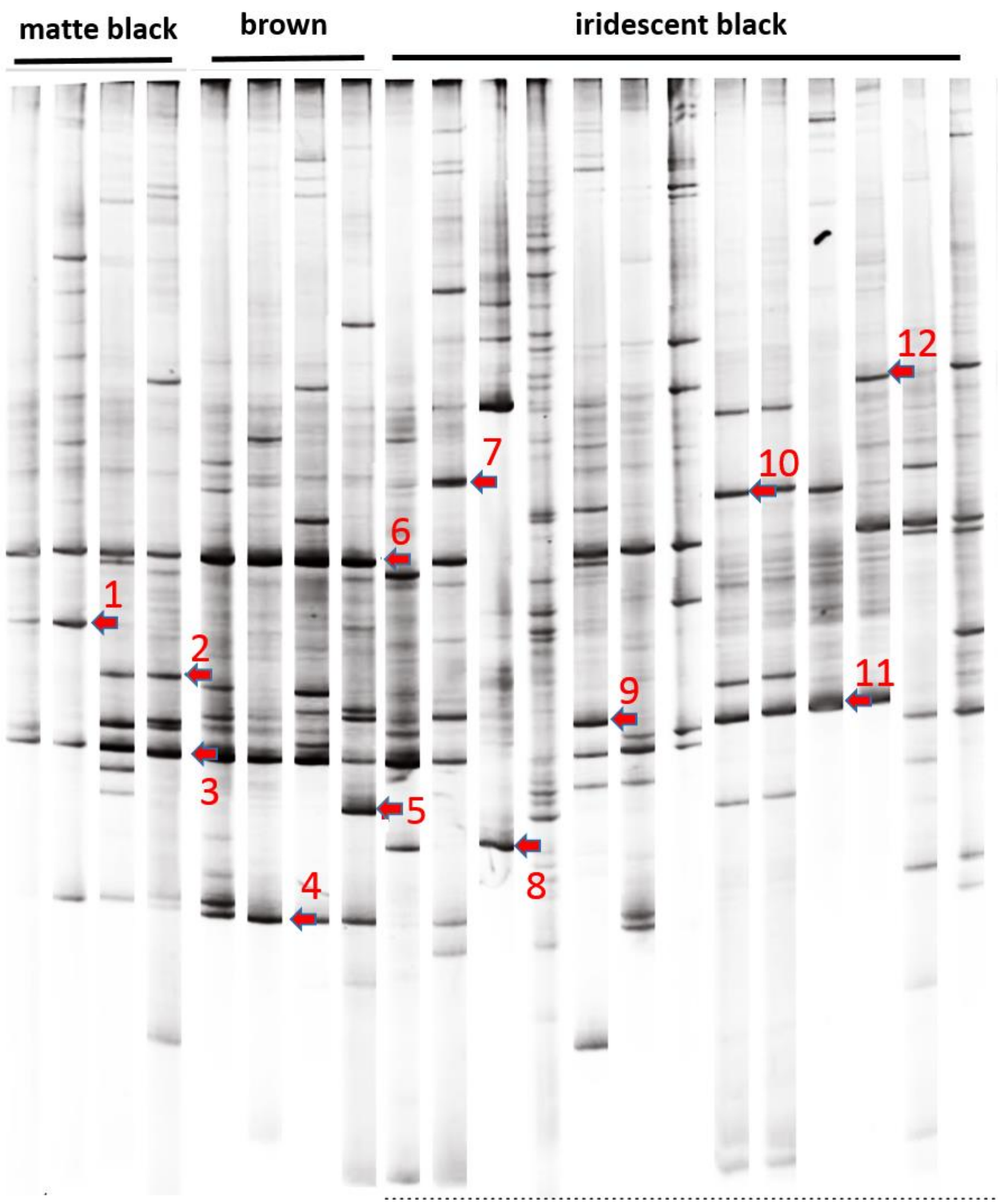

3. Supplementary Table S1

Table S1. Tukey´s HSD table comparing variance in White-shouldered Fairywren feather microbial load between sexes nested within plumage phenotype (black vs. brown). Significant effects (α = 0.05) are in bold.

Comparison	*diff*	*lwr*	*upr*	*p adj*
brown:F - black:F	-0.2970805	-1.7199809	1.12581989	0.9356326

black:M - black:F	-1.0365664	-2.0849429	0.01181014	0.0533178
brown:M - black F	-0.4379737	-2.0513911	1.17544370	0.8714122
black:M - brown:F	-0.7394859	-1.9177593	0.43878762	0.3224149
brown:M - brown:F	-0.1408932	-1.8415845	1.55979807	0.9954626
brown:M - black:M	0.5985927	-0.8038333	2.00101861	0.6371140

3. Supplementary Table S2

Table S2. Tukey´s HSD table comparing White-shouldered Fairywren feather microbial α-diversity between sexes nested within plumage phenotype (black vs. brown). Significant effects (α = 0.05) are in bold.

Comparison	diff	lwr	upr	p adj
brown:F - black:F	0.02159761	-0.07861954	0.1218147662	0.9266646
**black:M - black:F**	**0.12048107**	**0.04608913**	**0.1948730162**	**0.0013114**
brown:M - black F	-0.01481839	-0.16152128	0.1318845045	0.9914442
**black:M - brown:F**	**0.09888346**	**0.01540326**	**0.1823636618**	**0.0174056**
brown:M - brown:F	-0.03641600	-0.18793010	0.1150980968	0.9020603
brown:M - black:M	-0.13529946	-0.27111995	0.0005210271	0.0510755
